# Systematic search for putative new domain families in *Mycoplasma gallisepticum *genome

**DOI:** 10.1186/1756-0500-3-98

**Published:** 2010-04-12

**Authors:** Chilamakuri CS Reddy, Sane Sudha Rani, Bernard Offmann, R Sowdhamini

**Affiliations:** 1 Université de La Réunion, Equipe de Bioinformatique, Laboratoire de Biochimie et Génétique Moléculaire, 15 ave René Cassin, 97715 Saint Denis Messag Cedex 09, La Réunion, France; 2 National Centre for Biological Sciences, GKVK Campus, Bellary Road, 560065 Bangalore, India

## Abstract

**Background:**

Protein domains are the fundamental units of protein structure, function and evolution. The delineation of different domains in proteins is important for classification, understanding of structure, function and evolution. The delineation of protein domains within a polypeptide chain, namely at the genome scale, can be achieved in several ways but may remain problematic in many instances. Difficulties in identifying the domain content of a given sequence arise when the query sequence has no homologues with experimentally determined structure and searching against sequence domain databases also results in insignificant matches. Identification of domains under low sequence identity conditions and lack of structural homologues acquire a crucial importance especially at the genomic scale.

**Findings:**

We have developed a new method for the identification of domains in unassigned regions through indirect connections and scaled up its application to the analysis of 434 unassigned regions in 726 protein sequences of *Mycoplasma gallisepticum *genome. We could establish 71 new domain relationships and probable 63 putative new domain families through intermediate sequences in the unassigned regions, which importantly represent an overall 10% increase in PfamA domain annotation over the direct assignment in this genome.

**Conclusions:**

The systematic analysis of the unassigned regions in the *Mycoplasma gallisepticum *genome has provided some insight into the possible new domain relationships and putative new domain families. Further investigation of these predicted new domains may prove beneficial in improving the existing domain prediction algorithms.

## Background

Domain assignment to the protein sequences has paramount importance in the post genomic era. Protein domains are the structural, functional and evolutionary units of proteins. Study of proteins at the domain level has had a profound impact on the study of individual proteins. Experimental and/or computational methods can be used to identify domains in the given protein sequence. Classification databases such as the Dali Domain Dictionary[1], CATH[2], SCOP[3] and DIAL[4] employ structural data to locate and assign domains. Identification of domains at the sequence level depends on the detection of global and local sequence similarities between a given query sequence and domain sequences found in databases such as Pfam[5]. Due to high evolutionary divergence, it is not always possible to identify distantly related protein domains by sequence search techniques. The realization of additional domains in those circumstances can be tedious, involving manual intervention, but can lead to better understanding of overall biological function. We have recently introduced an automatic multi-step approach, PURE for recognizing such connections[6,7].

*Mycoplasma gallisepticum *causes chronic respiratory disease in chickens and other avian species. The infections result in considerable economic losses in poultry production. This pathogen has a small genome with 726 proteins [8], but only 498 protein sequences have known Pfam hits with 46% residue coverage [9]. The gap in the annotation of this genome emphasizes the need for further exploration for other methods for domain assignment from sequence. We have recently shown that it is possible to enhance prediction of domains in the unassigned regions by 25% through indirect connections in the class III adenylyl cyclase domain containing proteins [10]. Here, we demonstrate that this method can be scaled up for whole genome analysis, by taking *Mycoplasma gallisepticum *genome as a specific example.

## Results and Discussion

**Figure 1:**
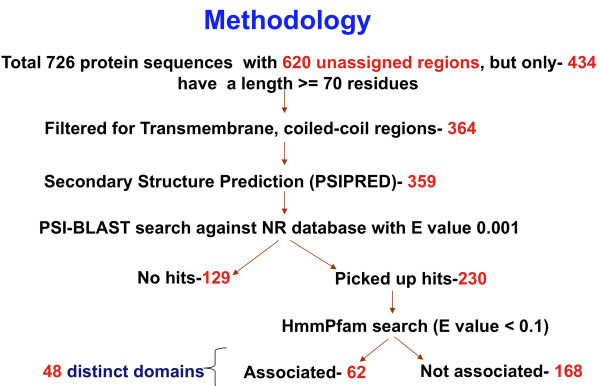
**Methodology of the approach and statistics**. Initially, 726 protein sequences were considered from *Mycoplasma gallisepticum *genome, which have 620 unassigned regions of different lengths. 434 unassigned regions are at least 70 residues long. Out of 434, only 364 passed through transmembrane and coiled coil filtering and 359 sequences after secondary structure filtering. The remaining unassigned regions (359) sequences were subject to PSI-BLAST searches, but only 230 unassigned regions picked up at least two hits. We extracted full-length sequences for each hit in PSI-BLAST and used for HMMpfam search. Here again, only 62 unassigned regions were associated indirectly with pre-existing domains which correspond to 48 different domain families.

The procedure that was followed in this study and overall results are described in Figure 1. Initially, there are 726 proteins in the *Mycoplasma gallisepticum *genome; domains were assigned to the genome with HMMpfam program of HMMER package [11] by search against Pfam 21 databases [5,12] with expectation value cut off of 0.01. Only PfamA families are used in HMMpfam search, since PfamB families were generated automatically and have no associated annotation, references and relatively low quality data than PfamA families [12]. The regions in protein sequences, which are associated with domains, are called 'assigned regions'; rest of the regions without PFAM domain assignment are called 'unassigned regions'. There were 620 unassigned regions in the 726 proteins. To avoid false positives in the similarity search, the unassigned regions with at least 70 residues long were considered. In our analysis, 434 unassigned regions have at least 70 residues long. Transmembrane, coiled-coils and low complexity regions were excluded and only 364 sequences passed through this filtering step. A further filter was placed to ensure adequate secondary structural content giving rise to 359 sequences. 15% cut-off on predicted structural content was chosen as the minimum value, consistent with our earlier work [13]. Only 230 out of 359 unassigned regions picked up at least two hits in PSI-BLAST search; full-length sequences for all the non-self hits were obtained and used as query for HMMpfam search to assign domains to the sequences. Only 62 of 230 unassigned regions were associated with PfamA domains through homologous sequences and remaining 168 unassigned sequences failed to pick up domain associations. These 62 unassigned sequences correspond to 71 newly predicted domains out of which 58 were fully associated and 13 were partially associated. In the fully associated domains, at least 75% of Pfam domain should lie within the unassigned region, otherwise it is called as 'partially associated domain'.

These 71 newly predicted domains belong to 48 unique domain families, but among the newly predicted 48 unique domain families, only 22 unique domains were initially not present in the *Mycoplasma gallisepticum *genome; for the remaining 26 domains, one or more copies already exist in the genome. Among the new predictions, 22 domains appear specific to *M. gallisepticum *genome, only 15 domains were present in other *Mycoplasmataceae *members and remaining 7 domains are unique to the entire *Mycoplasmataceae *family members (Table 1).

**Table 1:** Newly predicted domains in the *Mycoplasma gallisepticum *genome.

**S.No**.	Domain Name	Description	Full	Partial
1.	AAA	ATPase family associated with various cellular activities	NP_853502.1 = 9-182	-

2.	Anticodon_1	Anticodon-binding domain. This domain is found valyl and leucyl tRNA synthetases. It binds to the anticodon of the tRNA.	NP_852939.1 = 397-590 NP_852935.1 = 55-218 NP_853215.1 = 620-850	-

3.	ATP-synt_ab_C	ATP synthase alpha/beta chain, C terminal domain.	NP_853478.1 = 140-221	-

4.	ATP-synt_ab_N	ATP synthase alpha/beta family, beta-barrel domain	NP_853438.1 = 4-126 NP_853439.1 = 2-125	-

5.	BPD_transp_1	Binding-protein-dependent transport system inner membrane component.	NP_853029.1 = 53-260 NP_853249.1 = 59-232	-

6.	CHASE-3 #	Cyclases/Histidine kinases Associated Sensory Extracellular) present in diverse receptor-like proteins with histidine kinase and nucleotide cyclase domains	NP_853387.1 = 55-582	-

7.	DUF-30	Domain of Unknown Function 30	NP_853479.1 = 370-770	-

8.	DUF-31	Domain of Unknown Function 31	NP_853440.1 = 220-317 NP_853441.1 = 233-337 NP_853488.1 = 220-340	-

9.	LMP $	LMP repeated region. Found in the LMP group of surface-located membrane proteins of Mycoplasma hominis.	NP_853333.1 = 1260-1320, 1420-1580, 1600-1760	-

10.	Ferritin $	Ferritin-like domain is one of the major non-haem iron storage proteins in animals, plants, and microorganisms	NP_852976.1 = 5-143	-

11.	GMP_synt_C $	GMP synthase C terminal domain.	NP_852801.1 = 220-275	-

12.	Helicase_C	Helicase conserved C-terminal domain. Found in a wide variety of helicases and helicase related proteins.	NP_852813.1 = 440-530 NP_853467.1 = 660-730	-

13.	HGTP_anticodon	Anticodon binding domain. tRNA synthetases, or tRNA ligases are involved in protein synthesis. This domain is found in histidyl, glycyl, threonyl and prolyl tRNA synthetases.	NP_852966.1 = 342-423	-

14.	HHH #	The helix-hairpin-helix DNA-binding motif is found to be duplicated in the central domain of RuvA.	NP_853482.1 = 589-619 NP_853386.1 = 63-92, 98-127	-

15.	HTH_11 #	Helix-turn-helix domain present in a wide variety of proteins.	NP_853136.1 = 28-73	-

16.	HTH_12 #	Ribonuclease R winged-helix domain. Found found at the amino terminus of Ribonuclease R and a number of presumed transcriptional regulatory proteins from archaea.	NP_853240.1 = 38-89	-

17.	S1	The S1 domain occurs in a wide range of RNA associated proteins. It is structurally similar to cold shock protein which binds nucleic acids. The S1 domain has an OB-fold structure.	NP_852895.1 = 140-210	-

18.	KH_1	The K homology (KH) domain was first identified in the human heterogeneous nuclear ribonucleoprotein (hnRNP) K. It is a domain of around 70 amino acids that is present in a wide variety of quite diverse nucleic acid-binding proteins.	NP_852895.1 = 333-393	-

19.	Lactamase_B	Metallo-beta-lactamase superfamily.	NP_852865.1 = 40-248	-

20.	RMMBL	RNA-metabolising metallo-beta-lactamase.	NP_852865.1 = 320-360	-

21.	Lipoprotein_17	Lipoprotein associated domain.	NP_852799.1 = 49-160	-

22.	MatE	Multi Antimicrobial Extrusion (MATE) family function as drug/sodium antiporters.	NP_853011.1 = 364-530	-

23.	Methyltransf_3 $	O-methyltransferase	NP_852906.1 = 7-185	-

24.	MFS_1 $	Major Facilitator Superfamily	NP_852970.1 = 480-922	-

25.	NusB $	The NusB protein is involved in the regulation of rRNA biosynthesis by transcriptional antitermination.	NP_853291.1 = 13-130	-

26.	Peptidase_M23 $	Peptidase family M23	NP_853190.1 = 484-657	-

27.	PGM_PMM_IV $	Phosphoglucomutase/phosphomannomutae, C-terminal domain	NP_853364.1 = 481-550	-

28.	PTS_EIIB	phosphotransferase system, EIIB	NP_853326.1 = 47-85	-

29.	SBP_bac_1 $	Bacterial extracellular solute-binding protein	NP_852821.1 = 1-385	NP_852814.1 = 6-181

30.	Sigma70_r1_1 #	Sigma-70 factor, region 1.1.	NP_853171.1 = 288-342	-

31.	Sigma70_r1_2 $	Sigma-70 factor, region 1.2	NP_853171.1 = 357-398	-

32.	Sigma70_r4_2 $	Sigma-70, region 4	NP_852863.1 = 120-170	-

33.	TGS $	ThrRS, GTPase, and SpoT domain.	NP_852968.1 = 417-487	-

34.	THUMP $	thiouridine synthases, methylases and PSUSs domain.	NP_853282.1 = 78-170	-

35.	Transketolase_C	The C-terminal domain of transketolase has been proposed as a regulatory molecule binding site	NP_852812.1 = 530-641 NP_853134.1 = 138-262	-

36.	tRNA_anti	OB-fold nucleic acid binding domain	NP_852876.1 = 230-310	-

37.	VapD_N #	Virulence-associated protein D	NP_853458.1 = 7-49	-

38.	Lipoprotein_X	Mycoplasma MG185/MG260 protein.	-	NP_852988.1 = 247-404 NP_852899.1 = 257-414

39.	Lipoprotein_10	Putative mycoplasma lipoprotein, C-terminal region	NP_852988.1 = 444-563	-

40.	DEAD	Members of this family include the DEAD and DEAH box helicases. Helicases are involved in unwinding nucleic acids.	-	NP_852877.1 = 596-722

41.	ABC_membrane	ABC transporter transmembrane region.	-	NP_852786.1 = 2-126

42.	ABC_tran	ABC transporter	-	NP_853051.1 = 317-467

43.	DUF258	Domain of Unknown Function 258	-	NP_853404.1 = 7-104

44.	GTP_EFTU		-	NP_853200.1 = 68-151

45.	RecO #	Recombination protein O	-	NP_853174.1 = 1-74

46.	SBP_bac_5 $		-	NP_853298.1 = 461-889

47.	Transposase_mut	Transposase, Mutator family	-	NP_852891.1 = 6-108 NP_853257.1 = 16-83 NP_852883.1 = 2-121

48.	HNH $	HNH endonuclease	NP_853456.1 = 650-708	-

Among probable new domains, some of the them are first time identified in *Mycoplasma gallisepticum *genome (indicated by $ or #). Among those unique domains, some are not even present in other *Mycoplasmas *(indicated by #), while some of them are present in other *Mycoplasmas *(indicated by $). Second column indicates the name of the domain, third column a brief description about the domain and fourth and fifth columns indicate the kind of association which may be full association (we refer 'fully associated' when at least 75% of unassigned region is indirectly aligned with domain region) or partial association (we call partial associated when at most 75% of unassigned region is indirectly aligned with domain region). Under full and partial columns, we indicate the protein id and start and end residue number of probable new domain.

# Unique domain, this domain was not presently identified in entire *Mycoplasmataceae *family

$ Unique domain but present in other *Mycoplasmataceae *family members

To validate the newly predicted domains, we generated multiple sequence alignments using CLUSTALW program [14]. Inputs for multiple sequence alignment are the unassigned sequence and the representative sequences of the newly assigned domain obtained from Pfam database. In most cases, only few family-specific signature residues are conserved, suggesting extreme levels of evolutionary divergence from classical members of such Pfam families.

The number of newly predicted domains was substantial; it raises an interesting question: why were these domains not annotated in the initial search? It is likely because of the poor sequences identities between query and hit. Though sequence analysis-based remote-homology detection approaches, such as Hidden Markov Models (HMMs), are powerful tools, these methods often face limitations due to poor sequence similarities and non-uniform sequence dispersion in protein sequence space. Several interesting approaches have been employed in different ways to detect remotely related proteins; one such approach is based on the intermediate sequences. Intermediate sequence procedure substantially increases the ability to recognize the distant evolutionary relationships[15].

There is relatively large number (63) of unassigned regions, which has picked up at least five homologues but not associated with any PfamA domain (Additional file 1: Supplemental Table S1). We examined below few examples of these regions, which can be regarded as potentially putative new domain families. Search against PfamB profile HMM of Pfam24 database showed that 20 unassigned regions, where putative new domain families were predicted by us, were also associated with at least one PfamB domain (Additional file 2: Supplemental Table S2). However, about two-third (43 out of 63 unassigned regions) neither have a hit in the PfamA database nor in the PfamB database. These 43 regions may indicate potential new domain families, which are yet to be annotated in the Pfam database (Additional file 2: Supplemental Table S2).

**Figure 2:**
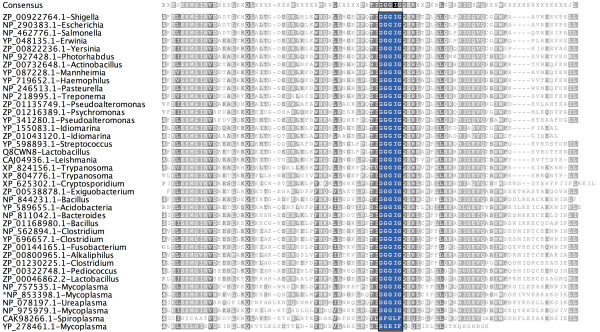
**Multiple sequence alignment of unassigned region (NP_853398.1.-242-329) and its homologues obtained in PSI-BLAST search**. Unassigned region indicated by '*' mark and consensus sequence is shown on the top of the alignment. Species name is given along with sequence ids. The highly conserved GGGIG motif is highlighted.

**Figure 3:**
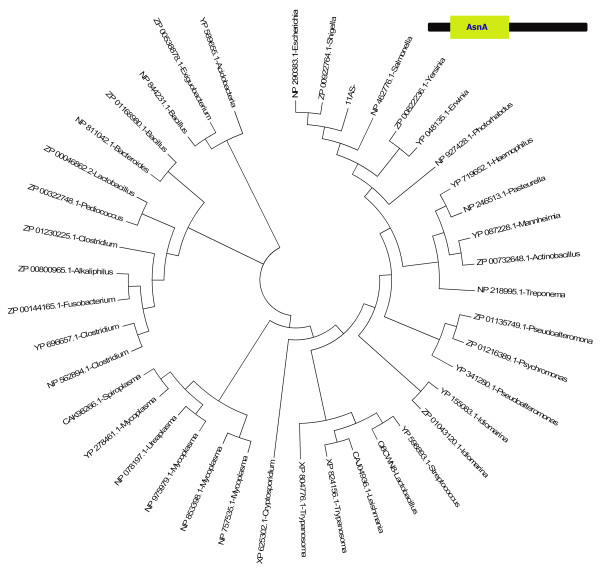
**Phylogenetic tree of homologues obtained in the PSI-BLAST search**. Domain architecture is shown on the top-right. All the homologues have identical domain architecture with amino-terminal AsnA domain. The mode of deriving phylogenetic trees is as described in Methods.

The NP_853398.1 protein (Figure 2, Figure 3) sequence, which is 329 residues long, has a single asparagine synthetase (AsnA) domain from 5 to 241 residues and one C-terminus unassigned region from 242 to 329. When the unassigned region of this protein (242-329) was analyzed, based on the intermediate sequences using the methodology described above, 106 similar sequences were identified in the PSI-BLAST search and these hit sequences were from both prokaryotes and eukaryotes. In the HMMpfam search, however, it was not associated with any PfamA domain, rather it was associated with PfamB_3316 domain. This unassigned region has about 62% predicted secondary structural content with 5 helices and 3 β-strands. More interestingly, the predicted β-3α-β-α-β-α secondary structure pattern is conserved in all the homologous sequences. All the homologous sequences have similar domain architecture. The crystal structure of *E.coli *asparagine synthetase also showed the presence of this small subdomain[16]. Aspartate--ammonia ligase (asparagine synthetase) catalyses the conversion of L-aspartate to L-asparagine in the presence of ATP and ammonia. AsnA structure revealed that AsnA structure is similar to that of the catalytic domain of yeast aspartyl-tRNA synthetase despite low sequence similarity. These enzymes have a common reaction mechanism that implies the formation of an aminoacyl-adenylate intermediate. The cluster of highly conserved residues (GGGIG) motif plays an important role in the formation of a cavity which can accommodate bound ATP in aspartyl-tRNA synthetase[16]. Since this motif is conserved in the newly predicted putative domain, it may play an important role in the ligand binding.

**Figure 4:**
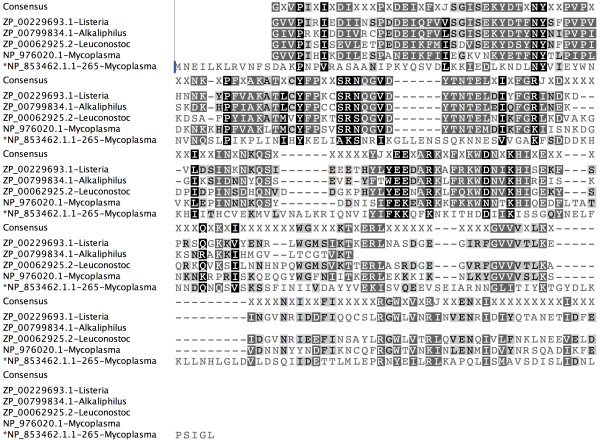
Multiple sequence alignment of unassigned region NP_853462.1.1-265 indicated by '*' in the alignment and its homologues obtained in the PSI-BLAST search. Consensus sequence showed on the top the alignment and the species names given along with sequence ids.

In another example, NP_853462.1 protein has an unassigned region (1-265) in the N-terminal region followed by a C-terminal existing Peptidase_S8 domain. All the PSI-BLAST hits were from prokaryotes. The alignment (Figure 4) and conservation of secondary structures in this region suggests the existence of functionally unidentified domain that could augment the basic peptidase activity. It is also interesting to note that all the homologous sequences have similar domain architectures.

**Figure 5:**
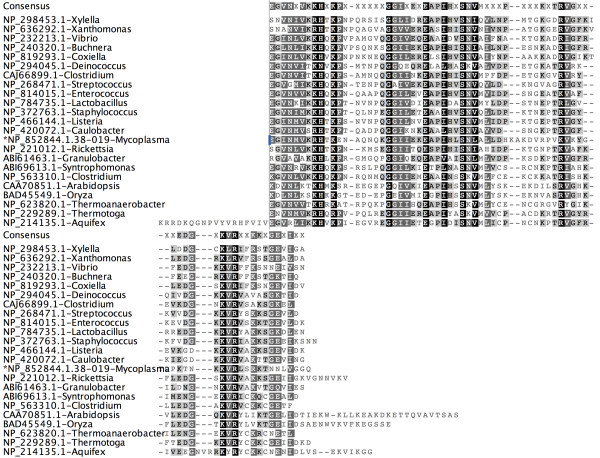
Multiple sequence alignment of unassigned region NP_852844.1.39-109, (indicated by '*' in the alignment) and its homologues obtained in the PSI-BLAST search. Consensus sequence is shown on the top of the alignment.

In another protein NP_852844.1, we had analysed an unassigned region from residues 38 to 109 and the gene product already was associated with KOW domain at the N-terminus (from 5 to 37 residues). 109 homologues could be identified by PSI-BLAST and all homologues belong to prokaryotic organisms (Figure 5). All the homologous sequences have similar predicted secondary structure content. Most of the homologues also have similar domain architecture with N-terminal KOW motif and C- terminus as an unassigned region. KOW motif is only about 35 residues long and links a bacterial transcription factor with ribosomal proteins[17]. The presence of conserved residues, with twice the size of KOW motif at the C-terminal region, suggests the functional role of C-domain in additionally stabilizing the oligomeric assemblies and thereby perhaps contributing to improved efficiency of protein expression.

The results presented in this paper, are based on Pfam21 database with 8957 protein domain families; updated version of Pfam database (Pfam23) has 10340 domain families. Comparison of 71 newly predicted domains in the 62 unassigned regions to the updated Pfam23 database revealed that 67 domains, out of 71, still remain unassigned in the Pfam23 database. Among four Pfam23 annotations, three are the same as our earlier prediction and one annotation differs from our prediction. Comparison of our results with conserved domain database (CDD v2.16)[18] revealed that 34 out of 71 unassigned regions remained unassigned in the CDD database also, whereas remaining 37 regions are annotated as domains. Out of these 37 annotated domains in the CDD database, 25 domains are the same as predicted by our method described above. Assignments which differ from the new Pfam database (Pfam23) and CDD (seeming false positives and false negatives) were analyzed further. 12 out of 71 PURE predicted domains differ when compared to CDD database (Table 2); These differences could arise due to inherent differences in the methodologies of CDD and Pfam database construction. When compared with Pfam23 database, out of 71 PURE predictions, 67 still remain unassigned and three agreed up on the PURE predictions. But, there is one disagreement: 124 residue-long protein NP_853458.1 was full-length unassigned sequence and PURE method assigned 'VapD_N' domain with E value of 0.006. In the Pfam23 database, 'CRISPR_Cas2' domain is assigned to the sequence with E value of 3.4 e^-47^. This could be due to the short length of 'VapD_N' (40 residues long) domain alignment and the borderline E-value (0.006 and a cut-off 0.01). Moreover, the 'CRISPR_Cas2' (PF09827) domain family is defined only in Pfam23, but not in Pfam21 database.

**Table 2:** Comparison of PURE predicted domains with CDD predicted domains

S.No	Seq id	PURE	CDD
1	NP_852935.1	antocodon_1 - 55-218	MetG - 10-199

2	NP_853333.1	LMP - 1260-1320 LMP - 1420-1580 LMP - 1600-1760	SbcC - 1253-1856

3	NP_853467.1	Helicase_C - 660-730	Type I site-specific restriction-modification system- 2-1018

4	NP_852865.1	Lactamase_B - 40-248 RMMBL - 320-360	mRNA degradation ribonucleases-23-594

5	NP_853011.1	MatE - 364-530	NorM - 127-558

6	NP_852970.1	MFS_1 - 480-992	SecD - 359-574

7	NP_853282.1	THUMP - 78-170	PseudoU_synth - 112-167

8	NP_853404.1	DUF_258 - 7-104	YlqF - 12-174

9	NP_853458.1	VapD_N	

Sequence id in the second column, PURE predicted domains in the third column, domain name followed by starting and ending residue of the domain shown. In the last column CDD predicted domains name of the domain followed by domain range shown.

The nature of sequence/domain searches is such that the databases are constantly going through updates and it is inevitable that our new findings might appear obsolete due to the constant updates of robust databases such as Pfam and CDD. Where there is concurrence with the newer version of a database, they serve to validate the approach. Where there is still new information obtained from PURE approach, this clearly suggests the value and novelty of the protocol due to the early realization of additional domains. When the newer entries are substantially high, this is very encouraging for the development of the approach suggesting that this has promise for discovering hitherto unidentified domains.

## Conclusions

Here, we present the results of the application of a new method for domain identification to full genome of an avian pathogen *Mycoplasma gallisepticum*. In spite of filters, such as evolutionary conservation and high predicted structural content, about 20% of orphan proteins contained in this genome could be annotated with a known functional domain using our approach. Interestingly, our analysis revealed several meaningful alignments, which could relate to as yet functionally unidentified set of domains. This could be very useful as a starting subset for further functional screening in wet lab experiments. Several improvements of the methodology will be addressed in future. Furthermore, cross-genome comparisons of the results from our procedure between *Mycoplasma gallisepticum *and other *Mycoplasma *species are currently being investigated.

## Methodology

Complete protein sequences of *Mycoplasma gallisepticum *(Strain R) were obtained from National centre for Biotechnology Information website[19].

### Extraction of Unassigned regions

*Mycoplasma gallisepticum *protein sequences were scanned against a dataset of Hidden Markov Models (HMMs) obtained from the PfamA database (Pfam version 21.0)[5] which consists of 8957 families, employing the HMMpfam of the HMMER suite[11], with E-value threshold set to 0.1. Sequences or sequence regions, which are not associated with any domain in the above search, were considered as unassigned regions.

### Filtering of Unassigned regions

The unassigned sequences thus obtained were subjected to different filtering steps.

a. To avoid false positives in the PSI-BLAST [20] search, we considered only unassigned sequences with at least 70 residues long.

b. Transmembrane regions were excluded by using HMMTOP [21] and coiled-coil regions by using COILS[22] from the unassigned sequences. The above steps carried out to avoid non-specific hits in the PSI-BLAST [20] search.

c. Standalone version of protein secondary-structure prediction program PSIPRED[23] was used to predict the α-helical, β-strand and coil (loop) content of different amino acids of unassigned regions. We employ 15% predicted secondary structural content as the minimum value, consistent with earlier work [13].

### PSI-BLAST searches

The unassigned sequences, which have fulfilled the filtering criteria, were used to query non-redundant sequence database [24] employing PSI-BLAST [20], with low complexity filter turned on (E value cut off 0.001), to obtain homologues.

### HMMpfam search

Only regions of homologues that aligned well with the query sequence were obtained from PSI-BLAST output to scan against a dataset of Hidden Markov Models (HMMs), which were obtained from the PfamA database (Pfam version 21.0)[5] which consists of 8957 families, employing HMMpfam of HMMER suite [11].

The indirect association of query sequence through homologous sequences with HMMs in the Pfam database gave rise to the definitions of full and partially associated domains. At least 75% of HMM should indirectly align with query to be considered as a fully associated domain and rest were considered as partially associated domains.

### Multiple sequence alignments

Multiple sequence alignments of the query unassigned region and seed sequences of predicted domains which were obtained from Pfam[9] were performed using CLUSTALW program [14]. Multiple sequence alignments of the query unassigned region and hits in the PSI-BLAST were also performed when unassigned regions, where hits were obtained by PSI-BLAST search, but not in HMMpfam search. When necessary, alignments were optimized by manual editing. Phylogenetic trees were calculated using Neighbor-Joining (NJ) method [25]. Phylogenetic tree was plotted using MEGA package [26].

## Competing interests

The authors declare that they have no competing interests.

## Authors' contributions

CSRC has developed algorithms and codes used to identify domains and performed analysis on the whole genome of *Mycoplasma gallisepticum*. SSR compared the results with other domain databases. BO helped to device the experiments and to interpret analysis. RS devised the whole strategy for domain identification using indirect approach and supervised the work of CSRC. CSRC wrote the first draft of the manuscript. Both BO and RS contributed to the writing and critical reading of the manuscript. All authors read and approved the final manuscript.

## Supplementary Material

Additional file 1 **Supplemental Table ST1 Predicted putative new domain families**. In our analysis, some of the unassigned regions shared homologues (at least 5 hits in PSI-BLAST search), but failed to associate with any Pfam domain in Hmmpfam search. We performed multiple sequence alignments to assess sequence conservation. 63 unassigned regions showed good conservation which may indicate putative new domain family. In the table, second column indicates the protein-id and third column (UR = Unassigned Region) indicates the start and end points of unassigned regions. Fourth column indicates the percentage secondary structural content in the unassigned region by using PSIPRED program. Fifth column indicates the total number of hits in the PSI-BLAST search and the sixth column indicates the number of PDB hits in PSI-BLAST search. Seventh column indicates the number of different species among the PSI-BLAST hits and eighth column indicates number of transmembrane helices which indicates whether protein is likely to be membrane-bound or not. Click here for file

Additional file 2 **Supplemental Table ST1 PfamB associations in the unassigned regions**. 63 unassigned sequences were used to search against PfamB database (Pfam 24), only 20 unassigned sequences were associated with at least one PfamB domain. Unassigned sequence id along with start and end residues are showed in column two, PfamB start and end residue numbers are shown in column three, PfamB ids are shown in column four and the Expectation values with which PfamB domains associated are shown in the last column. Click here for file
