# Supplementary material for: Systematic search for putative new domain families in Mycoplasma gallisepticum genome
Source: BMC Res Notes. 2010 Apr 12;3:98. doi: 10.1186/1756-0500-3-98 (PMC2865477; doi:10.1186/1756-0500-3-98)
Supplement: Additional file 1 — Supplemental Table ST1 Predicted putative new domain families. In our analysis, some of the unassigned regions shared homologues (at least 5 hits in PSI-BLAST search), but failed to associate with any Pfam domain in Hmmpfam search. We performed multiple sequence alignments to assess sequence conservation. 63 unassigned regions showed good conservation which may indicate putative new domain family. In the table, second column indicates the protein-id and third column (UR = Unassigned Region) indicates the start and end points of unassigned regions. Fourth column indicates the percentage secondary structural content in the unassigned region by using PSIPRED program. Fifth column indicates the total number of hits in the PSI-BLAST search and the sixth column indicates the number of PDB hits in PSI-BLAST search. Seventh column indicates the number of different species among the PSI-BLAST hits and eighth column indicates number of transmembrane helices which indicates whether protein is likely to be membrane-bound or not. [file 1756-0500-3-98-S1.PDF]

Additional files

Supplementary Table1: Putative new domains. In our analysis, some of the unassigned regions shared homologues (at least 5 hits in PSI-BLAST search), but failed to associate with any PFAM domain in Hmmpfam search. We performed multiple sequence alignments to assess sequence conservation. 63 unassigned regions showed good conservation which may indicate putative new domain family. In the table, second column indicates the protein-id and third column (UR = Unassigned Region) indicates the start and end point of unassigned regions. Fourth column indicates the percentage of secondary structural content in the unassigned region by using PSIPRED program. Fifth column indicates the total number of hits in the PSIBLAST search and the sixth column indicates the number of PDB hits in PSI-BLAST search. Seventh column indicates the number of different species among the PSI-BLAST hits and eighth column indicates number of transmembrane helices which indicates whether protein is likely to be membrane-bound or not.

SNo.	Protein ID	Unassigned Region	%Secondary Structure	No.of Homologues	Number of Structural Homologues	Number of different Species	Number of Transmembrane helices
1	NP_852957.1	1-99	65	250	0	188	0
2	NP_852957.1	377-480	63	250	0	177	0
3	NP_853020.1	638-762	74	250	1	63	4
4	NP_853253.1	468-596	74	94	0	75	0
5	NP_853264.1	273-345	39	250	8	141	0
6	NP_853357.1	1-156	57	250	0	168	0
7	NP_852803.1	1-439	59	20	0	8	2
8	NP_852912.1	1-719	57	53	0	0	0
9	NP_852964.1	1-921	48	38	0	3	6
10	NP_852978.1	182-268	91	31	0	3	0
11	NP_852980.1	1-78	35	58	0	3	2
12	NP_852981.1	1-83	33	57	0	3	2
13	NP_852982.1	1-81	28	58	0	3	2
14	NP_852983.1	1-90	34	58	0	3	2
15	NP_852984.1	1-99	26	58	0	3	2
16	NP_852985.1	174-260	91	31	0	3	0
17	NP_852986.1	1-80	33	58	0	3	2
18	NP_853047.1	1-169	69	19	0	3	0
19	NP_853120.1	1-75	42	58	0	3	2
20	NP_853121.1	1-81	35	58	0	3	2

21	NP_853124.1	1-78	37	58	0	3	2
22	NP_853125.1	1-74	37	58	0	3	2
23	NP_853126.1	1-106	23	58	0	3	2
24	NP_853127.1	1-73	38	57	0	3	2
25	NP_853210.1	184-270	91	31	0	3	0
26	NP_853477.1	1-204	52	31	0	2	0
27	NP_853486.1	1-691	36	36	0	2	2
28	NP_853489.1	1-453	44	34	0	2	0
29	NP_852788.1	328-425	42	250	0	124	0
30	NP_852802.1	246-372	63	250	0	166	0
31	NP_852819.1	164-255	59	186	0	130	0
32	NP_852820.1	241-509	58	250	0	188	0
33	NP_852820.1	592-665	81	250	4	175	0
34	NP_852844.1	38-109	50	250	1	218	0
35	NP_852876.1	1301-1501	58	250	0	158	0
36	NP_852904.1	571-668	67	250	0	177	0
37	NP_852908.1	1-74	52	250	0	161	0
38	NP_852922.1	1-71	57	250	4	181	0
39	NP_852940.1	300-458	57	250	1	182	0
40	NP_852968.1	158-247	84	250	0	164	0
41	NP_853092.1	1-87	54	250	4	79	0
42	NP_853104.1	1-1060	74	192	0	12	0
43	NP_853107.1	1-770	62	43	0	19	0
44	NP_853160.1	188-288	52	167	1	133	0
45	NP_853173.1	286-368	59	250	2	199	2
46	NP_853175.1	125-230	63	250	1	198	0
47	NP_853225.1	71-144	74	250	2	205	0
48	NP_853227.1	1-134	61	250	0	166	0
49	NP_853270.1	1-163	62	223	0	138	6
50	NP_853274.1	613-717	62	250	1	161	0
51	NP_853281.1	163-255	63	204	0	143	0
52	NP_853286.1	341-457	69	14	0	9	0
53	NP_853296.1	371-444	51	81	0	52	0
54	NP_853302.1	1-137	69	250	0	142	2
55	NP_853335.1	281-402	63	250	0	194	2
56	NP_853356.1	101-192	61	89	0	57	0
57	NP_853371.1	224-325	66	250	0	149	0
58	NP_853398.1	242-329	61	106	1	68	0
59	NP_853405.1	335-428	73	250	0	80	0
60	NP_853471.1	1-375	56	51	0	2	2
61	NP_853504.1	362-440	53	250	0	183	0
62	NP_853462.1	1-265	64	6	0	6	0
63	NP_852791.1	1-149	54	9	0	9	4
