# Supplementary material for: Systematic search for putative new domain families in Mycoplasma gallisepticum genome
Source: BMC Res Notes. 2010 Apr 12;3:98. doi: 10.1186/1756-0500-3-98 (PMC2865477; doi:10.1186/1756-0500-3-98)
Supplement: Additional file 2 — Supplemental Table ST1 PfamB associations in the unassigned regions. 63 unassigned sequences were used to search against PfamB database (Pfam 24), only 20 unassigned sequences were associated with at least one PfamB domain. Unassigned sequence id along with start and end residues are showed in column two, PfamB start and end residue numbers are shown in column three, PfamB ids are shown in column four and the Expectation values with which PfamB domains associated are shown in the last column. [file 1756-0500-3-98-S2.DOC]

**Table ST2:** PfamB associations in the unassigned regions. 63 unassigned sequences were used to search against PfamB database (Pfam 24), only 20 unassigned sequences associated with at least one PfamB domain. Unassigned sequence id along with start and end residues showed in the column two, PfamB start and end residue number shown in the column three, PfamB ids shown in the column four and the Expectation values with which PfamB domains associated shown in the last column.

| S.No. | Sequence id | Start residue-End residue | PfamB ID | Expectation value |
| --- | --- | --- | --- | --- |
|  | NP_852904.1.571-668 | 3-97  2-98 | PB007586  PB000831 | 4e-07  3.8e-05 |
|  | NP_852957.1.1-99 | 1-99  1-99  27-98 | PB000831  PB007586  PB017978 | 4.1e-19  2.1e-07  2.9e-05 |
|  | NP_852964.1.1-921 | 8-391 | PB005370 | 3.8e-49 |
|  | NP_852980.1.1-78 | 3-68 | PB007501 | 0.00067 |
|  | NP_852981.1.1-83 | 3-67  22-80  17-76  10-69  15-68  20-80  22-63  2-80  22-79  23-69  15-78  19-73  7-60  23-79 | PB007501  PB010136  PB018845  PB004087  PB018572  PB001640  PB018291  PB004744  PB013256  PB018490  PB006000  PB009450  PB005160  PB009902 | 0.00022  0.00044  0.00011  0.00018  0.00046  5.3e-05  0.00027  2.2e-05  0.00028  0.00049  0.00076  0.001  0.001  0.00076 |
|  | NP_852983.1.1-90 | 8-84  2-82  4-73  7-75  15-85  18-73  15-83  20-75  19-88  20-79  17-75  14-80  21-75  22-73 | PB012986  PB004744  PB007501  PB004087  PB009961  PB002671  PB019325  PB018291  PB009767  PB018104  PB012999  PB006000  PB013256  PB006333 | 0.00098  0.00014  1.8e-06  5e-06  9e-05  0.00065  0.00025  5.2e-07  7.1e-06  0.00023  0.00062  0.0003  0.00051  0.001 |
|  | NP_852984.1.1-99 | 8-92  16-96  11-64  19-97  21-79  16-93  17-75  18-94  20-76  9-96  5-65  14-92  18-95  19-73  17-95  21-75  18-96  8-97  21-75  16-99  22-91  23-96  23-98  23-69  23-97 | PB012986  PB009961  PB000827  PB009767  PB018104  PB019325  PB018845  PB016851  PB018291  PB004087  PB005160  PB006000  PB009450  PB002671  PB018134  PB013256  PB003907  PB005207  PB013734  PB018572  PB001640  PB014100  PB006333  PB018490  PB009902 | 0.00016  1.7e-06  0.00011  9.6e-08  3.4e-05  5.6e-05  0.00013  0.00068  7.7e-09  1.2e-07  3.8e-06  1.1e-05  1.8e-05  0.00011  0.00019  2.5e-05  7.1e-05  0.00016  0.00029  4.7e-05  0.0001  0.00061  5.8e-06  3.6e-05  0.00029 |
|  | NP_852986.1.1-80 | 15-78  4-73  17-76  16-62  8-65  22-60  19-76  23-61 | PB009961  PB007501  PB001640  PB018572  PB004087  PB018291  PB001024  PB018490 | 0.001  0.0006  0.00027  0.00072  0.00039  0.00039  0.00036  0.00052 |
|  | NP_853120.1.1-75 | 19-54 | PB018291 | 0.00013 |
|  | NP_853121.1.1-81 | 4-75  12-78  3-79  7-66  14-73  17-77  6-60  4-77 | PB007501  PB016763  PB012325  PB012999  PB009961  PB001640  PB004087  PB004744 | 0.00018  0.00092  0.00013  0.00024  0.00029  0.0004  0.00048  0.00054 |
|  | NP_853124.1.1-78 | 3-65  10-63  21-57 | PB007501  PB004087  PB018291 | 0.00025  0.00088  0.00028 |
|  | NP_853125.1.1-74 | 3-68  14-73  22-73  20-71  7-69  17-69  15-69  18-74  4-62  22-73  16-73  20-67  2-70  23-65  17-70  20-71  23-69  22-73  23-72  23-71  7-71 | PB007501  PB009961  PB010136  PB001640  PB004087  PB009767  PB018572  PB018845  PB005160  PB013256  PB006000  PB008447  PB004744  PB018291  PB009450  PB001024  PB018490  PB006333  PB009902  PB014100  PB005207 | 0.00044  0.00096  0.00014  0.00014  9.6e-06  0.00048  6.5e-05  0.00049  2.1e-05  0.00028  0.00069  0.00092  3.7e-05  0.00011  0.00017  0.00063  2.6e-05  0.00016  0.00023  0.00047  0.00046 |
|  | NP_853126.1.1-106 | 11-94  15-92  19-97  17-94  14-93  16-102  21-86  19-95  18-96  14-96  23-100  10-89 | PB004087  PB012999  PB009767  PB003907  PB006000  PB016763  PB018291  PB009450  PB018134  PB001024  PB009902  PB005207 | 2.3e-06  2.6e-05  1.9e-05  0.00016  8.9e-05  0.00028  0.00083  9.8e-05  0.00047  8.9e-05  9.3e-05  0.00025 |
|  | NP_853175.1.125-230 | 17-105 | PB002899 | 1.4e-06 |
|  | NP_853286.1.341-457 | 42-78  42-110  53-93 | PB012318  PB016297  PB013215 | 3e-08  6.2e-12  0.00041 |
|  | NP_853356.1.101-192 | 2-90 | PB005829 | 4.3e-07 |
|  | NP_853371.1.224-325 | 1-47 | PB000097 | 1.4e-09 |
|  | NP_853477.1.1-204 | 9-192 | PB001981 | 1.9e-54 |
|  | NP_853486.1.1-691 | 6-148  32-144  10-133  42-140  35-149  5-124  47-182  38-166  232-663 | PB004087  PB009450  PB001024  PB005319  PB018285  PB005160  PB013256  PB009767  PB001981 | 6.6e-06  0.00016  5.3e-06  0.00011  0.00065  2.9e-06  1.1e-06  2.1e-05  4.2e-140 |
|  | NP_853489.1.1-453 | 23-441 | PB001981 | 5.7e-144 |
